# Modelling of amorphous cellulose depolymerisation by cellulases, parametric studies and optimisation

**DOI:** 10.1016/j.bej.2015.10.017

**Published:** 2016-01-15

**Authors:** Hongxing Niu, Nilay Shah, Cleo Kontoravdi

**Affiliations:** Centre for Process Systems Engineering, Department of Chemical Engineering, Imperial College London, South Kensington Campus, London SW7 2AZ, England, UK

**Keywords:** Cellulose, Cellulase, Modelling, Uncertainty, Kinetic parameters, Optimisation

## Abstract

Improved understanding of heterogeneous cellulose hydrolysis by cellulases is the basis for optimising enzymatic catalysis-based cellulosic biorefineries. A detailed mechanistic model is developed to describe the dynamic adsorption/desorption and synergistic chain-end scissions of cellulases (endoglucanase, exoglucanase, and β-glucosidase) upon amorphous cellulose. The model can predict evolutions of the chain lengths of insoluble cellulose polymers and production of soluble sugars during hydrolysis. Simultaneously, a modelling framework for uncertainty analysis is built based on a quasi-Monte-Carlo method and global sensitivity analysis, which can systematically identify key parameters, help refine the model and improve its identifiability. The model, initially comprising 27 parameters, is found to be over-parameterized with structural and practical identification problems under usual operating conditions (low enzyme loadings). The parameter estimation problem is therefore mathematically ill posed. The framework allows us, on the one hand, to identify a subset of 13 crucial parameters, of which more accurate confidence intervals are estimated using a given experimental dataset, and, on the other hand, to overcome the identification problems. The model’s predictive capability is checked against an independent set of experimental data. Finally, the optimal composition of cellulases cocktail is obtained by model-based optimisation both for enzymatic hydrolysis and for the process of simultaneous saccharification and fermentation.

## Highlights

• A mechanistic model for heterogeneous cellulose hydrolysis by cellulases.
• A modeling framework for uncertainty analysis, model reduction and refinement.
• The parameters were estimated.
• Composition of cellulases cocktail was optimized using the model.

## Nomenclature

C: accessible binding sites on cellulose unoccupied by enzymes (mmol sites/L)
cov(ϴ): covariance matrix of estimated parameters
$D_{\theta_{1,...,k}},D$: variances in model outputs associated with simultaneous changes in the parameters ϴ_1,…,p_ and in all the parameters, respectively
*diag*: diagonal elements of a matrix
DP( = *N*): initial polymerization degree of cellulose substrate
*E *= *en*, *ex*, *bg*: E = englucanase, exoglucanse, beta-glucosidase, respectively
*E*’ = *en*, *ex*: E’ = englucanase, exoglucanse
*E*_a_: activation energy (kJ/mol)
*E*_load_: enzyme loading (g/L)
*E*_l, f_: concentration of free enzyme in liquid phase (g/L)
*E*_l, t_: concentration of total enzymein liquid phase (g/L)
*E*_s, f_: concentration of free enzyme in solid phase (g/L)
*E*_s,t_: concentration of total enzyme in solid phase (g/L)
$\langle E^{E}⊕G_{i}\rangle$: substrate-enzyme complex (g enzyme/L)
Fa: fraction of accessible β-glucosidic bonds
G1, G2, G3: concentration of glucose, cellobiose, cellotriose (mmol/L), respectively
Gi: concentration of cellulose polymer with polymerization degree i(mmol/L)
$I_{{GI}^{E}},I_{{G2}^{E\prime }},I_{{G3}^{E\prime }}$: inhibition constant of glucose, cellobiose, cellotriose (g/L), respectively
J(ϴ): sum of squares of residuals between simulations and measurements
$K_{{ad}^{E}}$: adsorption equilibrium constant (L/mmol sites)
$K_{{dis}^{E}}$: equilibrium constant of enzymatic hydrolysis (mmol β-glucosidic bonds/L)
*K*^E^: “apparent” reaction constant (103 mmol/(g enzyme × h))
k^*E^: “intrinsic” reaction constant (103 mmol/(g enzyme × h))
*k*_f_: adsorptionrate constant (L/(mmol sites × h))
*k*_r_: desorptionrate constant (h^−1^)
M^E^: enzyme molecular weight (g/mmol)
m, n, p: number of model variables, data points, parameters, respectively
$R_{e_{i}}$: relative values between first-order and total effect sensitivity indices
R(ϴ_i_, ϴ_j_): correlation coefficient between the estimated parameters ϴ_i_ and ϴ_j_
r_Gi_: production rate of G_i_ (mmol/(L × h))
$S_{\theta_{i}},{S_{\theta_{i}}}^{tot}$: first-order, total effect sensitivity indices with respect to parameter i, respectively
*W*_i_: weighting matrix
$Y_{G_{123}}/G_{N}$: conversion of cellulose *G*_N_ to soluble sugars (% C/C)
$y_{\text{i}},\hat{y}\left({\text{t}}_{\text{i}},\theta \right)$: measured variables, estimated variable values, respectively

**Greek letters**
α_t_: significance level for *t*-test
${2\alpha }^{E}$: number of cellobiose lattice occupied by one molecule of enzyme (mmol sites/mmol enzyme)
ϴ, ϴ^_lb_^, ϴ^ub^, ϴ_0_: parameter vector, lower and upper bounds, initial guesses, respectively
v: confidence intervals of parameters at αt significance level
ϴ_refer_: reference values for corresponding parameters, cited from literature
λ^E^: first order deactivation constant of enzyme (1/h)
δ^E^: binding capacity of substrate (mmol sites/mmol β-glucosidic bonds)
${\sigma^{2}}_{j}$: errorvariance of jth meansurement

## 1 Introduction

Enzymatic hydrolysis of cellulosic materials to produce reducing sugars has long been pursued for its potential for providing abundant food and energy resources. It is a multi-step process that takes place in a heterogeneous reaction system [1], in which insoluble cellulose is initially broken down at the solid-liquid interface (with enzyme adsorption/desorption) via the synergistic actions of endoglucanases ([EC 3.2.1.4]) and exoglucanases ([EC 3.2.1.91]). This initial degradation is accompanied by further liquid-phase hydrolysis of soluble intermediate products, i.e., short cellulose oligosaccharides and cellobiose, which are catalytically cleaved to produce glucose by the action of β-glucosidase ([EC 3.2.1.21]).

Mechanistic understanding of the overall hydrolysis system is certainly interesting for designing rational approaches for enzymatic hydrolysis and subsequent fermentation processes. However, the complexity of the system, which arises from the concerted action of several enzymes on a solid substrate/mixture in a heterogeneous system, makes experimental kinetic studies very difficult. Accordingly, although many models of enzymatic hydrolysis have been developed over the past decades, most of them are empirical correlations and data-driven and as a result are only applicable to specific cases/conditions [2], [3], [4], [5], [6], [7], [8]. Generally, they are: (1) simply lumping the different cellulolytic enzymes together as a single catalyst; (2) treating the cellulose mixture as a single bulk concentration; (3) simplifying the reaction system as a homogeneous one, i.e., without considering the enzyme adsorption onto and desorption from solid particles; (4) lacking analysis of model identifiability and parameter uncertainty [9]. These approaches are summarised in recent reviews of enzymatic hydrolysis of (ligno) cellulose [10], [11].

Efforts to propose mechanistic models have been made to enhance understanding of the enzymatic hydrolysis of cellulose [12], [13], [14], [15], [16], [17], [18], [19], [20], [21], [22], [23], [24]. However, they mainly lack thorough parametric studies and experimental validation. Consequently, the predictability of the models, especially for extrapolations, is still in doubt. At the same time, studies to investigate fundamental mechanisms of random hydrolysis (random chain scission) and processive hydrolysis (chain-end scission) of polymers have been carried out extensively using population balance modelling [25], [26], [27], [28], [29], [30]. Population balance modelling involves tracking the numbers of entities and behaviour of a population of particles based on the analysis of the behaviour of single particles in local conditions [31], [32]. The results provide clues to the underlying mechanisms of the enzymatic hydrolysis process of cellulose.

Following the above advances, this work develops a mechanistic depolymerisation (scission) model of enzymatic hydrolysis, taking into account the enzyme adsorption/desorption processes in this heterogeneous system. Furthermore, a systematic sensitivity analysis-based method is proposed for parametric studies, model reduction and verification with published experimental data. Finally, the model’s predictive capability is checked against an independent set of data and model-based optimisation studies are presented.

## 2 Model development

### 2.1 Model assumptions

**Fig. 1:**
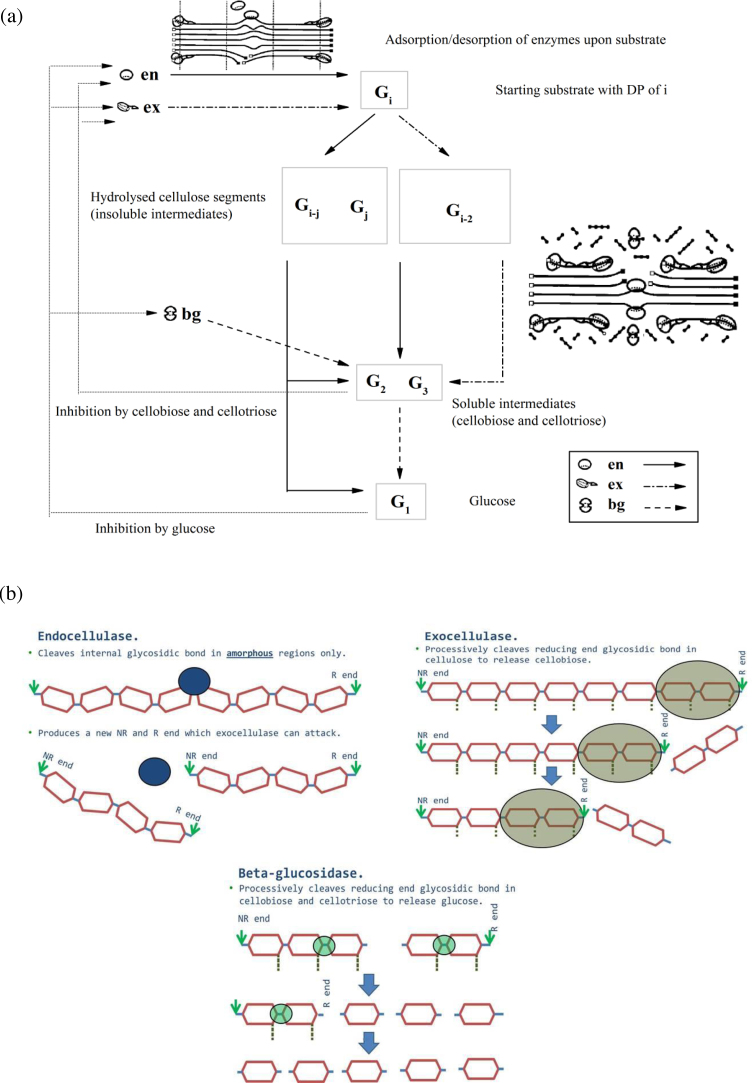
(a) Schematic representation of the concerted action by three cellulolytic enzymes en (endoglucanase), ex (cellobiohydrolase) and bg (beta-glucosidase) and hydrolysis producing different cellulose chain lengths. G_i_, starting substrate with DPi; G_i–j_, G_j_, and G_i-2_, hydrolysed cellulose segments of DP_i–j_, DP_j_, and DP_i-2_ (insoluble intermediate products); G_2_, cellobiose; G_3_, Cellotriose; G_1_, glucose. Action of en is represented by full arrow (→), of ex by dot-dash arrow (), and of bg by dashed arrow (). Feedback inhibition of cellotriose, cellobiose and glucose is shown. (b) Action modes of the three enzymes during cellulolysis: random scission by endocellulase (endoglucanase); chain-end processive scission by exocellulase (exoglucanase) releasing cellobiose; processive scission by beta-glucosidase on cellotriose and cellobiose producing glucose.

Model development is based on the following assumptions:

(1) There are several studies about how to modify and improve the various physical properties of cellulose as a substrate, such as particle size, fibre structure, accessibility, crystallinity index, and amorphicity index [24], [33], [34], [35], [36], [37], [38], [39]. In this work, for simplification, the substrate is assumed to be completely amorphous (non-crystalline) pure cellulose and be well ground into a very fine powder.
(2) The enzymatic hydrolysis takes place in a well-stirred tank reactor. As a result, there is no mass transfer limitation during enzymatic hydrolysis.
(3) The binding probability of enzyme to one polymer molecule is proportional to the molecule’s polymerization degree. In other words, the enzyme has equal accessibility to every β-glucosidic bond of the polymer [21], [22].
(4) The quasi-steady state approximation holds for any intermediate complex, i.e.,$\langle E^{E}⊕G_{i}\rangle E=en,ex,bg$.
(5) The loading of cellulases (i.e., total loading of the three core enzymes) is low. More specifically, the loading is no more than 15 mg/g-glucan and 750 mg/L. Accordingly, interaction/crowding effects between different kinds of enzymes are negligible during adsorption/desorption. The adsorption/desorption of each kind of enzyme is described separately and considered reversible.
(6) Cellulosic polymers with chain lengths over four are deemed to exist in solid phase and cannot dissolve into liquid. As shown by Fig. 1, solid particles (*DP *≥ 4) are depolymerized by endoglucanases and exoglucanases, while soluble shorter polymers (namely cellotriose and cellobiose) are exclusively cleaved by β-glucosidases. This is a reasonable assumption if one compares the enzyme specific activities between insoluble and soluble substrates. It has been found experimentally that both endoglucanases and exoglucanases have relatively low catalytic activities upon soluble sugars compared with those upon solid cellulose [11].
(7) The enzymes are inhibited by soluble sugars (Fig. 1) non-competitively; the inhibition of glucose increases proportionally to its concentration raised to the power of 3 [6]; additionally, enzyme activity decreases exponentially with time [40].

### 2.2 Dynamic adsorption/desorption of cellulases

The reaction of the enzyme-substrate complex is initiated upon physical contact of endoglucanases and exoglucanases with the surface of an insoluble substrate. Equilibrium of enzyme adsorption/desorption is represented by the Langmuir isotherm model. The time required for equilibrium to be reached is relatively short compared to the hydrolysis time [20], [41], [42], [43]. Therefore, the adsorption/desorption is decoupled from the formation of the enzyme-substrate complex. The adsorption/desorption is represented by

(1) $$C+E^{E}{}_{l,t}\overset{{k^{E}}_{f}}{\underset{{k_{r}}^{E}}{⇆}}E^{E}{}_{s,t}and{K^{E}}_{ad}=\frac{{E^{E}}_{s,t}}{{E^{E}}_{l,t}C}=\frac{{k^{E}}_{f}}{{k^{E}}_{r}}$$

where *C* is the accessible binding sites onto the β-glucosidic bonds of cellulose uncovered by enzymes, *E^E^_l,t_* and *E^E^_s,t_* are the total concentrations of one type of enzyme in liquid phase and adsorbed onto solid phase respectively, $K^{E}{}_{ad}$ is the adsorption equilibrium constant, and *k^E^_f_* and *k^E^_r_* are adsorption and desorption rate constants, respectively.

The material balances of enzymes and accessible binding sites on cellulose are represented by Eq. (2) and Eq. (3), respectively.

(2) $$E^{E}{}_{\text{load}}=E^{E}{}_{l,t}+E^{E}{}_{s,t}$$

(3) $$\sigma^{E}\sum_{i=4}^{N}\left[(i-1)G_{i}\right]=C+2\alpha^{E}\frac{E^{E}{}_{s,t}}{M^{E}}$$

where *E^E^*_load_ is the loading of any type of enzyme, *σ^E^* is the binding capacity of β-glucosidic bonds, *G_i_* represents the cellulose with polymerization degree *i* (so the total concentration in terms of “mmol β-glucosidic bonds/L” is (*i *− 1) *G_i_*), 2α*^E^* is the number of cellobiose lattice occupied by one molecule of enzyme, and *M^E^* is the enzyme molecular weight.

By eliminating the term *C* from Eq. (1) and Eq. (3), it is deduced that

(4) $$E^{E}{}_{s,t}=\frac{\sigma^{E}\sum_{i=4}^{N}\left[(i-1)G_{i}\right]}{\frac{1}{K^{E}{}_{ad}E^{E}{}_{l,t}}+\frac{2\alpha^{E}}{M^{E}}}$$

Furthermore, by eliminating the term *E^E^_l,t_* using Eq. (2), Eq. (4) is transformed into

(5) $$\frac{2\alpha^{E}}{M^{E}}E^{E}{{}_{s,t}}^{2}-\{\sigma^{E}\sum_{i=4}^{N}\left[(i-1)G_{i}\right]+\frac{2\alpha^{E}}{M^{E}}E^{E}{}_{load}+\frac{1}{K_{ad}}\}E^{E}{}_{s,t}+\sigma^{E}\sum_{i=4}^{N}\left[(i-1)G_{i}\right]E^{E}{}_{load}=0$$

By solving the above quadratic equation, we obtain the real root, taking into account $0\leq E^{E}{}_{s,t}\leq \min(\sigma^{E}\sum_{i=4}^{N}[(i-1)G_{i}],E^{E}{}_{\text{load}})$.

(Enzyme in solid phase *E^E^_s,t_* should be non-negative (≥0), not bigger than total enzyme loading *E^E^*_load_ or total binding capacity of substrate $\sigma^{E}\sum_{i=4}^{N}[(i-1)G_{i}]$).

(6) $$E^{E}{}_{s,t}=\frac{[\sigma^{E}\sum_{i=4}^{N}[(i-1)G_{i}]+\frac{2\alpha^{E}}{M^{E}}E^{E}{}_{load}+\frac{1}{K^{E}{}_{ad}}]-\sqrt{{\left\{\sigma^{E}\sum_{i=4}^{N}\left[(i-1)G_{i}\right]+\frac{2\alpha^{E}}{M_{E}}E^{E}{}_{load}+\frac{1}{K^{E}{}_{ad}}\right\}}^{2}-\frac{8\alpha^{E}}{M^{E}}\sigma^{E}\sum_{i=4}^{N}[(i-1)G_{i}]E^{E}{}_{load}}}{\frac{4\alpha^{E}}{M^{E}}}$$

*E*_load_ and *G_i_* may change over time, so

**Figure:**
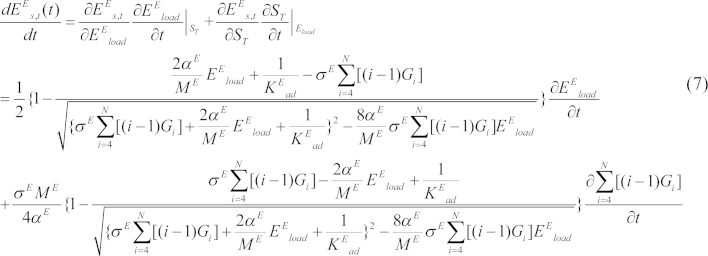

where $S_{T}=\sum_{i=4}^{N}\left[(i-1)G_{i}\right]$.

### 2.3 Enzymatic hydrolysis of cellulose

#### 2.3.1 By endoglucanases (en)

The random hydrolysis action by endoglucanases in solid phase is described by Michaelis–Menten kinetics  where *G_i_* represents the cellulose with polymerization degree *i*, ${E_{s,f}}^{en}$ is the “not complexed” endoglucanases in solid phase, $\langle E^{en}⊕G_{i}\rangle$ symbolizes the substrate-enzyme complex (in terms of “g endoglucanases/L”), and *k^en^* is reaction constant. Endoglucanases break β-glucosidic bonds of cellulose randomly, so the total solid substrate quantity is $\sum_{n=4}^{N}\left[(n-1)G_{n}\right]$. The equilibrium constant ${K_{dis}}^{en}$ is defined by [24],

(8) $${K_{dis}}^{en}=\frac{E_{s,}{{}_{f}}^{en}F_{a}(i-1)G_{i}}{\langle E^{en}⊕G_{i}\rangle }≅\frac{E_{s,}{{}_{f}}^{en}F_{a}\sum_{n=4}^{N}\left[(n-1)G_{n}\right]}{\sum_{n=4}^{N}\langle E^{en}⊕G_{n}\rangle }$$

where *F_a_* is the fraction of accessible β-glucosidic bonds. Accordingly, Eq. (9) is obtained by changing Eq. (8)

(9) $$\langle E^{en}⊕G_{i}\rangle ≅\frac{(i-1)G_{i}}{\sum_{n=4}^{N}\left[(n-1)G_{n}\right]}\sum_{n=4}^{N}\langle E^{en}⊕G_{n}\rangle$$

Using the enzyme mass balance (Eq. (10)), we can eliminate the term ${E_{s,f}}^{en}$ in Eq. (8) and obtain Eq. (11)

(10) $${E_{s,t}}^{en}={E_{s,f}}^{en}+\sum_{n=4}^{N}\langle E^{en}⊕G_{n}\rangle$$

(11) $$\sum_{n=4}^{N}\langle E^{en}⊕G_{i}\rangle =\frac{E_{s,}{{}_{t}}^{en}F_{a}\sum_{n=4}^{N}\left[(n-1)G_{n}\right]}{{K_{dis}}^{en}+F_{a}\sum_{n=4}^{N}\left[(n-1)G_{n}\right]}$$

From Eqs. (9) and (11), we further obtain

(12) $$\langle E^{en}⊕G_{i}\rangle =\frac{E_{s,}{{}_{t}}^{en}F_{a}(i-1)G_{i}}{{K_{dis}}^{en}+F_{a}\sum_{n=4}^{N}\left[(n-1)G_{n}\right]}$$

As shown in Eq. (12) the formation of one polymer-enzyme complex $\langle E^{en}⊕G_{i}\rangle$ is proportional to the product of total adsorbed enzyme ${E_{s,t}}^{en}$ and total accessible β-glucosidic bonds of the polymer *G_i_*.

The overall production rate of *G_i_* equals the contributions of *G_k_* (*k* *= i + *1, *i + *2,…*N*) minus the loss of *G_i_*, where *G_i_* is cleaved by endoglucanases with a probability of $\frac{2}{k-1}$ [21], [24], i.e.,

(13) $${r_{G_{i}}}^{en}=k^{en}(\sum_{k=i+1}^{N}\frac{2}{k-1}\langle E^{en}⊕G_{k}\rangle -\langle E^{en}⊕G_{i}\rangle )=\frac{k^{en}E_{s,}{{}_{t}}^{en}F_{a}[2\sum_{k=i+1}^{N}G_{k}-(i-1)G_{i}]}{{K_{dis}}^{en}+F_{a}\sum_{n=4}^{N}\left[(n-1)G_{n}\right]}i\geq 4$$

Additionally, the formation rate of soluble sugars dissolving into the liquid phase is obtained by

(14) $${r_{G_{j}}}^{en}=\frac{2k^{en}E_{s,}{{}_{t}}^{en}F_{a}\sum_{k=4}^{N}G_{k}}{{K_{dis}}^{en}+F_{a}\sum_{n=4}^{N}\left[(n-1)G_{n}\right]}j=1,2,3$$

#### 2.3.2 By exoglucanases (ex)

Processive hydrolysis by exoglucanases is represented by

$$G_{i}+{E_{s,f}}^{ex}\overset{{K_{dis}}^{ex}}{⇆}\langle E^{ex}⊕G_{i}\rangle \overset{k^{ex}}{\to }{E_{s,f}}^{ex}+G_{i}{}_{-2}+G_{2}(i\geq 4)$$

where ${E_{s,f}}^{ex}$ is the “not complexed” exoglucanases in solid phase, and $\langle E^{ex}⊕G_{i}\rangle$ symbolizes the substrate-enzyme complex (g exoglucanases/L). There are generally two functionally different kinds of exoglucanases, namely CBH-I and CBH-II, which processively cut off cellulosic polymers from reducing and non-reducing chain ends, respectively [44], [45]. The total enzyme concentration in this case is the sum of CBH-I and CBH-II assuming they have the same specific activity.

Similar to Eq. (8), the equilibrium constant is defined by

(15) $${K_{\text{dis}}}^{ex}=\frac{E_{s,}{{}_{f}}^{ex}F_{a}G_{i}}{\langle E^{ex}⊕G_{i}\rangle }≅\frac{E_{s,}{{}_{f}}^{ex}F_{a}\sum_{n=4}^{N}G_{n}}{\sum_{n=4}^{N}\langle E^{ex}⊕G_{n}\rangle }$$

Since exoglucanases act on cellulose by means of chain-end scission, the total substrate quantity in the solid phase is represented by $\sum_{n=4}^{N}G_{n}$ (compared with $\sum_{n=4}^{N}(n-1)G_{n}$ in Eq. (8)). The enzyme mass balance and substrate-enzyme complex concentration are obtained by Eq. (16) and Eq. (17), respectively

(16) $${E_{s,t}}^{ex}={E_{s,f}}^{ex}+\sum_{n=4}^{N}\langle E^{ex}⊕G_{n}\rangle$$

(17) $$\langle E^{ex}⊕G_{i}\rangle =\frac{E_{s,}{{}_{t}}^{ex}F_{a}G_{i}}{{K_{dis}}^{ex}+F_{a}\sum_{n=4}^{N}G_{n}}$$

The formation rate of $G_{i}$ equals the depolymerisation of $G_{i+2}$ minus that of $G_{i}$, as follows

(18) $${r_{G_{i}}}^{ex}=\frac{k^{ex}{E_{s,t}}^{Ex}F_{a}(G_{i+2}-G_{i})}{{K_{dis}}^{ex}+F_{a}\sum_{n=4}^{N}G_{n}}i\geq 4$$

and the production rates of cellotriose and cellobiose are obtained by Eq. (19) and Eq. (20), respectively

(19) $${r_{G_{3}}}^{ex}=\frac{k^{ex}{E_{s,t}}^{ex}F_{a}G_{5}}{{K_{\text{dis}}}^{ex}+F_{a}\sum_{n=4}^{N}G_{n}}$$

(20) $${r_{G_{2}}}^{ex}=\frac{k^{ex}{E_{s,t}}^{ex}F_{a}(\sum_{k=4}^{N}G_{k}+G_{4})}{{K_{\text{dis}}}^{ex}+F_{a}\sum_{n=4}^{N}G_{n}}$$

#### 2.3.3 By β-glucosidases (bg)

Processive hydrolysis of soluble sugars (i.e., cellotriose and cellobiose) by β-glucosidase is described by

$$G_{i}+{E_{l,f}}^{bg}\overset{{k_{dis}}^{bg}}{⇄}\langle E^{bg}G_{i}\rangle \overset{k^{bg}}{\to }{E_{l,f}}^{bg}+G_{i-1}+G_{1}\left(2\leq i\leq 3\right)$$

where ${E_{l,f}}^{bg}$ is the “not complexed” β-glucosidases in liquid phase, and $\langle E^{bg}⊕G_{i}\rangle$ symbolizes the substrate-enzyme complex (g β-glucosidases/L).

Like exoglucanases, β-glucosidases cleave off glucose from cellotriose and cellobiose in the way of processive hydrolysis. Similar to the analyses in Section 2.3.2, relevant equations are obtained and shown below

(21) $${K_{dis}}^{bg}=\frac{{E_{l,f}}^{bg}G_{i}}{\langle E^{bg}⊕G_{i}\rangle }≅\frac{{E_{l,f}}^{bg}\sum_{n=2}^{3}G_{n}}{\sum_{n=2}^{3}\langle E^{bg}⊕G_{n}\rangle }$$

(22) $${E_{l,t}}^{\text{bg}}={E_{l,f}}^{\text{bg}}+\sum_{n=2}^{3}\langle E^{\text{bg}}⊕G_{n}\rangle$$

(23) $$\langle E^{\text{bg}}⊕G_{i}\rangle =\frac{{E_{l,t}}^{\text{bg}}G_{i}}{{K_{\text{dis}}}^{\text{bg}}+\sum_{n=2}^{3}G_{n}}i=2,3$$

The production/consumption rates of cellotriose, cellobiose and glucose are calculated by Eq. (24), Eq. (25), and Eq. (26), respectively,

(24) $${r_{G_{3}}}^{\text{bg}}=-\frac{k^{\text{bg}}{E_{l,t}}^{\text{bg}}G_{3}}{{K_{\text{dis}}}^{\text{bg}}+\sum_{n=2}^{3}G_{n}}=-\frac{k^{\text{bg}}({E_{\text{load}}}^{\text{bg}}-{E_{s,t}}^{\text{bg}})G_{3}}{{K_{\text{dis}}}^{\text{bg}}+\sum_{n=2}^{3}G_{n}}$$

(25) $${r_{G_{2}}}^{\text{bg}}=\frac{k^{\text{bg}}({E_{\text{load}}}^{\text{bg}}-{E_{s,t}}^{\text{bg}})(G_{3}-G_{2})}{{K_{\text{dis}}}^{\text{bg}}+\sum_{n=2}^{3}G_{n}}$$

(26) $${r_{G_{1}}}^{\text{bg}}=\frac{k^{\text{bg}}({E_{\text{load}}}^{\text{bg}}-{E_{\text{s},\text{t}}}^{\text{bg}})\left(\overset{3}{\underset{k=1}{\sum }}G_{k}+G_{2}\right)}{K_{\text{dis}}^{\text{bg}}+\sum_{n=1}^{3}G_{n}}$$

#### 2.3.4 Decrease in enzyme activity

It is often observed that the effectiveness of cellulases is drastically reduced during hydrolysis. Until now, this phenomenon is not well understood and remains an open research question that merits further studies such as [46], [47]. In this work, the possible reasons for the rate reduction are hypothesised to be inhibition by soluble sugars (Fig. 1(a)) and loss of enzyme activity. Accordingly, the reaction constants change into Eqs. (27) and (28).

(27) $$k^{\text{E}\prime }=k^{*\text{E}\prime }\frac{I_{G_{3}}^{\text{E}\prime }}{I_{G_{3}}^{\text{E}\prime }+G_{3}}\frac{I_{G_{2}}^{\text{E}\prime }}{I_{G_{2}}^{\text{E}\prime }+G_{2}}\frac{I_{G_{1}}^{\text{E}\prime }}{I_{G_{1}}^{\text{E}\prime }+G_{1}^{3}}\text{E}\prime =\text{en},\text{ex}$$

(28) $$k^{\text{bg}}=k^{*\text{bg}}\frac{I_{G_{1}}^{\text{bg}}}{I_{G_{1}}^{\text{bg}}+G_{1}}$$

where ${I_{G_{1}}}^{E^{\prime }}$ (and ${I_{G_{1}}}^{bg}$), ${I_{G_{2}}}^{E^{\prime }}$, and ${I_{G_{3}}}^{E^{\prime }}$ are respectively inhibition constants of glucose, cellobiose, and cellotriose. $k^{*E^{\prime }}$ and $k^{*bg}$ represent “intrinsic” reaction constants. The inhibition is considered to be non-competitive and the effect of glucose is taken to increase proportionally to its concentration raised to the power of 3 [6].

Additionally, thanks to first order kinetics of enzyme deactivation [40], the remaining relative enzyme specific activities after *t* time are

(29) $$\exp(-\lambda^{\text{E}}\times t),\text{E}=en,ex,\text{bg}$$

where *λ*^E^ represent the first order deactivation constants.

### 2.4 Summary of mass balances

The overall formation rates of each species are obtained as follows

(30) $$\frac{dG_{1}(t)}{dt}={r_{G_{1}}}^{en}\exp(-\lambda^{en}\times t)+{r_{G_{1}}}^{bg}\exp(-\lambda^{bg}\times t)$$

(31) $$\frac{dG_{2}(t)}{dt}={r_{G_{2}}}^{en}\exp(-\lambda^{en}\times t)+{r_{G_{2}}}^{ex}\exp(-\lambda^{ex}\times t)+{r_{G_{2}}}^{bg}\exp(-\lambda^{bg}\times t)$$

(32) $$\frac{dG_{3}(t)}{dt}={r_{G_{3}}}^{en}\exp(-\lambda^{en}\times t)+{r_{G_{3}}}^{ex}\exp(-\lambda^{ex}\times t)+{r_{G_{3}}}^{bg}\exp(-\lambda^{bg}\times t)$$

(33) $$\frac{dG_{i}(t)}{dt}={r_{G_{i}}}^{en}\exp(-\lambda^{en}\times t)+{r_{G_{i}}}^{ex}\exp(-\lambda^{ex}\times t)i\geq 4$$

## 3 Computational methods

### 3.1 Parameter estimation

One set of data for non-crystalline cellulose hydrolysis is used for parameter estimation [6], in which Spezyme^CP^ (Genencor, lot no. 301-00348-257) had an average activity of 31.2 filter paper units (FPU)/mL and was diluted to 1 and 3 FPU by adding buffer solutions. Another set of data for phosphoric acid swollen cellulose hydrolysis was used to check the model’s predictive ability, where mixtures of different mole percentage of *Humicola insolens* endoglucanase and enxoglucanase (CBH II), and *Penicillium brasilianum β*-glucosidase were used to hydrolyze phosphoric acid swollen cellulose [66].

The values of parameters are iteratively adjusted to obtain the most accurate agreement between predictions and measurements, i.e., by minimizing the weighted sum of squares of residuals:

(34) $$\begin{array}{c} J\left(\theta \right)=\sum_{i=1}^{n}{\left[\hat{y}(t_{1},\theta )-y_{1}\right]}^{T}W_{1}\left[\hat{y}(t_{1},\theta )-y_{1}\right]\\ \text{s}.\text{t}.\theta^{\text{lb}}\leq \theta \leq \theta^{\text{ub}} \end{array}$$

In order to tackle some drawbacks in multimodal optimisation problems such as dependency on initial guesses, a hybrid solution strategy is employed, combining the genetic algorithm with the Nelder–Mead simplex search method [48].

Assuming that the error terms for each experiment are additive, independently/uncorrelated and identically distributed normally with zero mean and variance *σ^2^_j_*, the inverse co-variance matrix (i.e., weighting matrix) is [49].

(35) $$W_{\text{i}}=\text{diag}({\sigma_{1}}^{-2},{\sigma_{2}}^{-2},...,{\sigma_{m}}^{-2})$$

When maximum likelihood estimation is used, the covariance matrix of θ is obtained by linear approximation [50].

(36) $$\text{cov}\left(\theta \right)≅\frac{J\left(\theta \right)}{\text{mn}-p}{\left\{[{▽}_{\text{K}}y^{\text{T}}\left(t,\theta \right)]W{\left[{▽}_{\text{K}}y^{\text{T}}\left(t,\theta \right)\right]}^{\text{T}}\right\}}^{-1}$$

Then, the confidence intervals of θ at α_t_ significance level are

(37) $$\begin{array}{c} \theta_{1-\theta_{t}}=\theta \pm \sqrt{\text{diag}\left(\text{cov}\left(\theta \right)\right)}\times t_{1-\alpha_{t/2}}\left(\text{mn}-p\right)\\ s.t.0\leq \theta_{1-\alpha_{t}} \end{array}$$

Furthermore, the approximate correlation matrix of θ can be obtained, the ijth element of which is given by

(38) $$R\left(\theta_{\text{i}},\theta_{\text{j}}\right)=\frac{\text{cov}\left(\theta_{\text{i}},\theta_{\text{j}}\right)}{\sqrt{\sigma_{\theta_{\text{i}}}^{2}\sigma_{\theta_{\text{j}}}^{2}}}$$

The diagonal elements of the matrix are all unity and the off-diagonal elements are in the interval [−1,1].

### 3.2 Global sensitivity analysis

Global sensitivity analysis (GSA), specifically the Sobol’ GSA method, is used to further evaluate the identifiability of parameters, i.e., sensitivities of model outputs (y) to variations of model parameters θ [51], [52]. The temporal profile of sensitivities of outputs with respect to parameter variation is calculated in terms of the sensitivity indices below

(39) $$S_{\theta_{1,...,k}}=\frac{D_{\theta_{1,...,k}}}{D}$$

where $D_{\theta_{\mathrm{1,}...\mathrm{,k}}}$ and *D* are the variation in model outputs associated with changes in each parameter *θ*_1,…,p_ and in all parameters simultaneously, respectively.

The first-order indices,$S_{\theta_{i}}$, indicate the sensitivity with respect to one individual parameter without interactions, while higher order indices,$S_{\theta_{1,...,k}}$, represent the effect of interactions between the parameters. Total effect indices with respect to each parameter,${S_{\theta_{i}}}^{tot}$, are defined as

(40) $${S_{\theta_{i}}}^{\text{tot}}=S_{\theta_{i}}+\sum_{j\neq i}^{k}S_{\theta_{i,j}}+\sum_{j,l\neq i}^{k}S_{\theta_{i,j,l}}+\cdots +S_{\theta_{i,j,l,\cdots ,k}}$$

Additionally, a matrix of relative values calculated by Eq. (41) indicates levels of parameter interactions, i.e., the closer the values are to one, the lower the interactions.

(41) $$R_{\theta_{i}}=\frac{S_{\theta_{i}}}{{S_{\theta_{i}}}^{\text{tot}}}$$

### 3.3 Model-based optimisation

As an optimisation criterion for cellulose hydrolysis, the percent conversion to soluble sugars in terms of % C/C is defined as

(42) $$Y_{G_{123/G_{N}}}=100\frac{G_{1}+{2G}_{2}+{3G}_{3}}{N\times G_{N}}$$

Process performance is compared with that of a simultaneous saccharification and fermentation (SSF) process [53], [54], [55], [56], [57], [58]. For this purpose, we consider an engineered *Geobacillus thermoglucosidasius* strain TM242 which can produce ethanol with an inoculum of 0.15 g DCW/L. Through systematic metabolic network reduction [59], a fermentation model (based on five main macro-metabolic reactions) is integrated with the above hydrolysis model to describe the SSF process [60].

By calculating percent conversion $Y_{G_{123}/G_{N}}$ for a batch process of the enzymatic hydrolysis and a batch SSF under different compositions of the three core enzymes, respectively, we can determine what compositions are optimal in either case with regard to cellulose conversion to soluble sugars. In the both processes, initial cellulose concentrations are set to 50 g/L.

## 4 Results and discussion

### 4.1 Parameter uncertainty and preliminary estimation

**Fig. 2:**
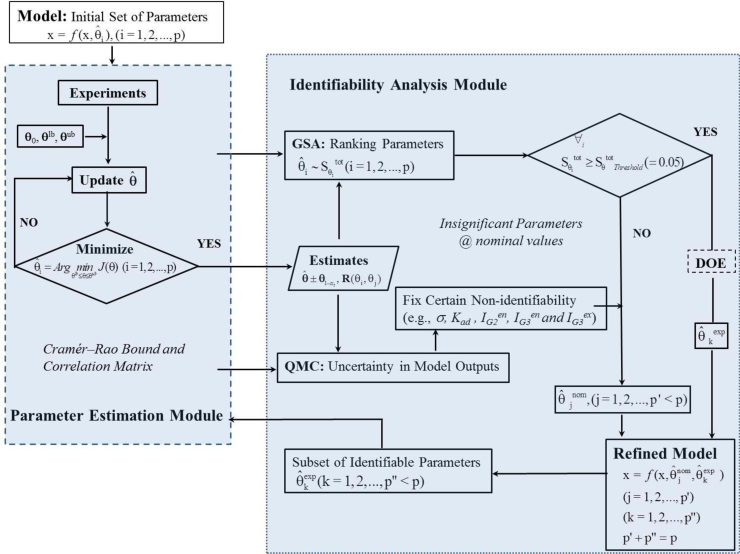
Flow chart of parameter estimation, uncertainty analysis, and model refinement. DOE: design of experiments; GSA: global sensitivity analysis; QMC: quasi-Monte-Carlo simulations.

Uncertainty analysis assesses the confidence in modelling results (including parameter estimates and model outputs/predictions) by quantification of the propagation of various sources of errors in the model input and design. The errors may originate from two sources: one is the quality and amount of data used to develop the model (each measurement is associated with a measurement noise and the system is usually observed partially); another is the model structure, which may not be a perfect representation of the real system. As shown in Eqs. (36)–(38), parameter uncertainty in terms of confidence intervals and correlation matrix can be estimated from the calculation of the Fischer information matrix based on local sensitivity analysis. Additionally, model prediction uncertainty will be investigated in Section 4.2 by two related methods: quasi Monte-Carlo (QMC) simulation using Sobol' sequences and global sensitivity analysis using the Sobol’ method (GSA). Fig. 2 depicts the framework used in this study.

One set of data for non-crystalline cellulose hydrolysis was used for assessing model fits and parameter estimation [6]. The activity of an enzyme in solution is proportional to its protein concentration [61] and 1PFU is approximated to 2 mg protein [62], [63], [64], [65]. The components of Spezyme^CP^ are assumed to be 12% w/w endoglucanases, 80% w/w exoglucanases, 4% w/w β-glucosidases, and other enzymes [1], [66], [67]. The molecular weights are 43 kDa, 65 kDa, and 110 kDa for endoglucanases, exoglucanases, and β-glucosidases, respectively [1], [66], [67].

The model includes 27 parameters in total, i.e., k^*E^, *K*_dis_^E^, *σ*^E^, *K*_ad_^E^, *I*_G1_^E^, *I*_G2_^E′^, *I*_G3_^E′^ *λ*^E^, 2*α*^E^ (*E* *=* *en, ex, bg*; *E*′ *=* *en*, *ex*), *F_a_* and *DP* of cellulose. For detailed definitions of the parameters, please refer to the nomenclature section. Initially, after GSA analysis was performed, eight non-significant parameters were set at their nominal values: reported values of *F_a_* and *DP* were used, i.e., 0.12 and 152, respectively [11]; *λ*^E^ values were fixed at 0.0122 h^−1^ [40], [67], [68]; and 2α^E^ were assumed to be the values of 43, 65, 110 for endoglucanases, exoglucanases, and β-glucosidases, respectively [69], [70]. As a result, there were 19 parameters left for estimation.

**Fig. 3:**
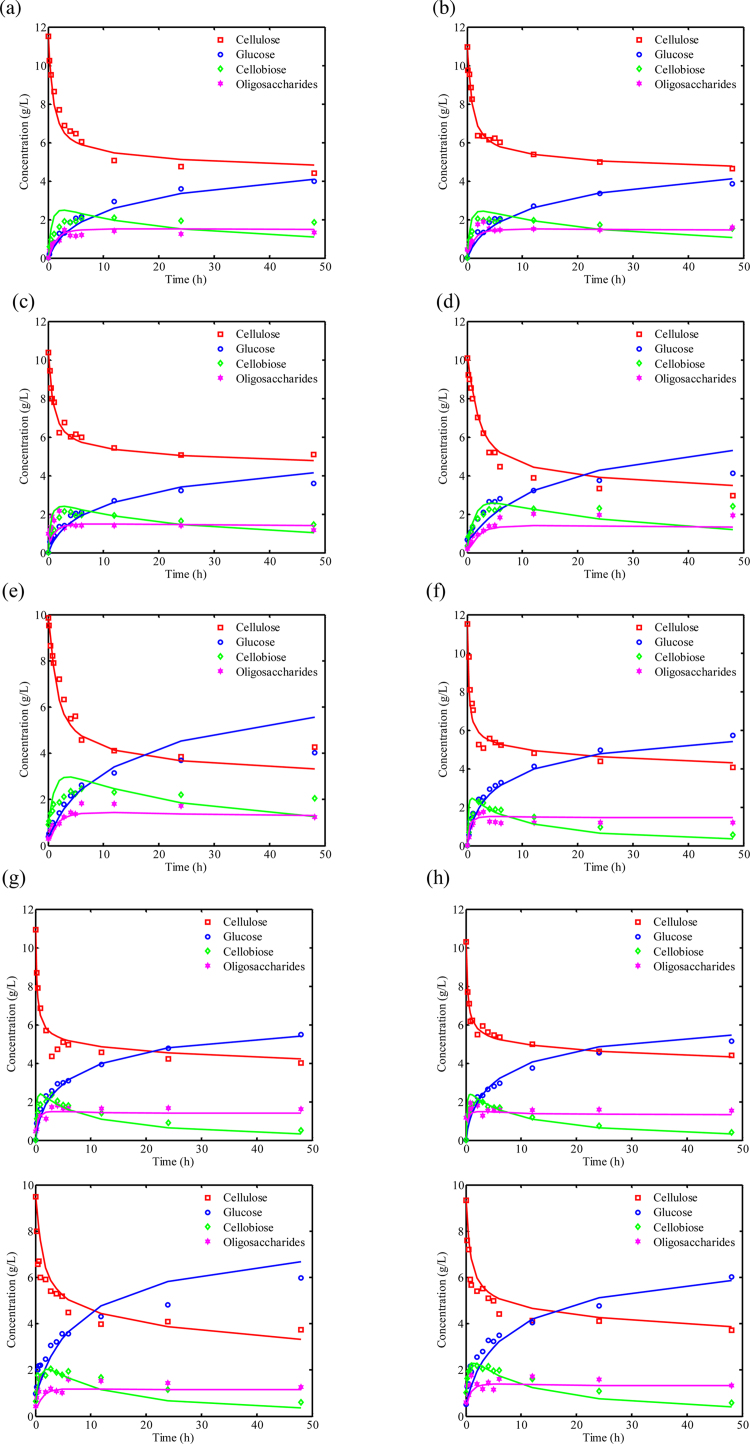
Experimental data and model predictions of non-crystalline cellulose hydrolysis, including the time profiles of insoluble cellulose, glucose, cellobiose, and oligosaccharides (G_3_–G_6_). Experimental data are represented as discrete points while model simulations are depicted as continuous lines. Cellulases loadings: 1 FPU/g-glucan in processes of (a)–(d); 3 FPU/g-glucan in processes of (e)–(h). Starting substrates: only non-crystalline cellulose in (a) and (e); non-crystalline cellulose with 5% (w/w) cello-oligosaccharides in (b) and (f); non-crystalline cellulose with 5% (w/w) glucose in (c) and (g); non-crystalline cellulose with 5% (w/w) cellobiose in (d) and (h). (Experimental data from Peri et al. [6])

As shown in Table 1, lower and upper bounds for constrained minimization (Eq. (34)) are set with reference to literature [1], [5], [6], [24], [11], [45], [62], [63], [71], [72], [73], [74], [75]. θ_0_ are initial guesses for the parameters. Preliminary parameter values estimated using this methodology and their 95% confidence intervals are summarized in Table 1. As illustrated in Fig. 3 depicting the dynamic concentration profiles of non-crystalline cellulose, glucose, cellobiose, and oligosaccharides for hydrolysis processes under different conditions [6], the model simulation results are in adequate agreement with the experimental data. Generally, a typical biphasic pattern is observed in every process, i.e., a faster initial rate of hydrolysis, which progressively slows down possibly due to product inhibition and enzyme deactivation [10], [11], [45].

**Fig. 4:**
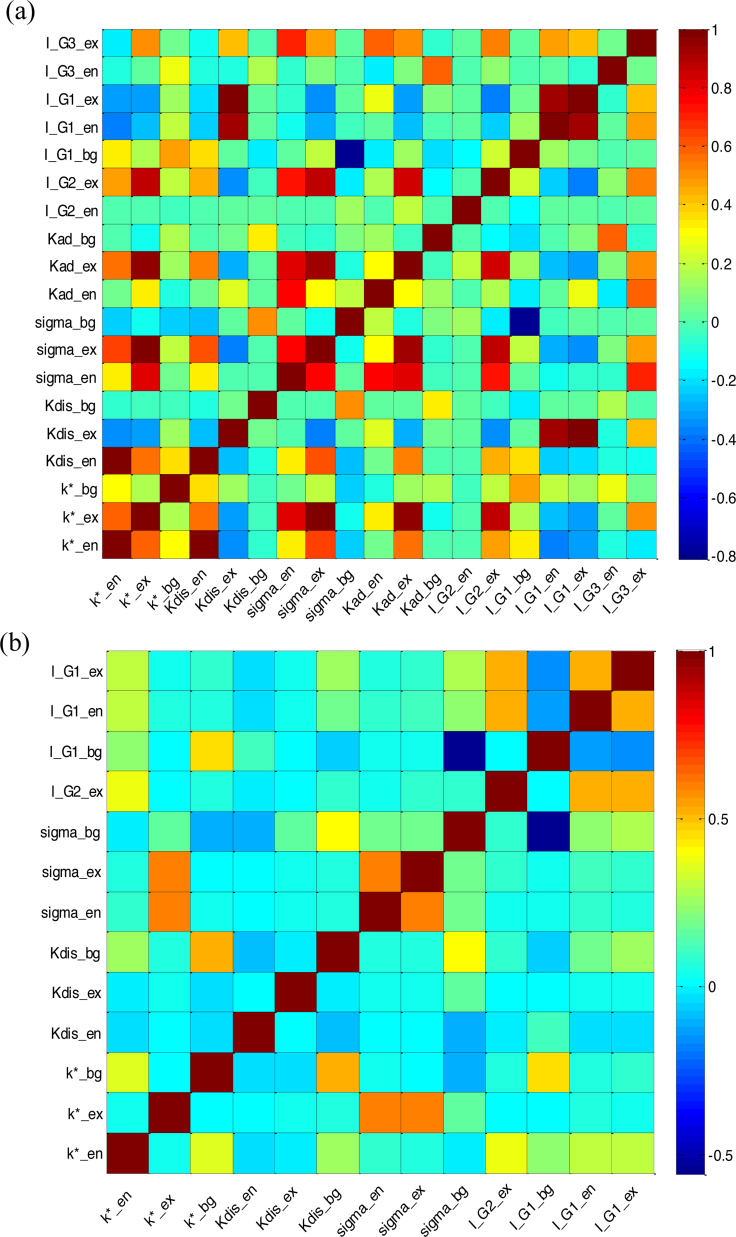
Correlation matrix of the identified parameters. (a), preliminary estimation, dim θ = 19; (b), refined estimation, dim θ = 13.

Moreover, the covariance matrix obtained by Eq. (36) allows analysis of the extent of correlation between the parameters (Eq. (38)). The correlation coefficients are shown in Fig. 4(a). High correlation coefficients (the absolute value of off-diagonal elements >0.7) are occasionally found between some parameters and the ratios of confidence intervals to means of certain parameters, especially *σ*^E^, implied by Table 1 are rather high, indicating the estimates are highly correlated and may be inaccurate. One reason is, to a large extent, the model structure, such as over parameterization, which is often encountered in the modelling of biorefinery processes [9], [76], [77]. Another possible reason would be poor experimental design/information content of data. In the following section, the model structure and prediction uncertainty are analysed by means of QMC and GSA to improve identifiability and reliability.

### 4.2 Prediction uncertainty, parameter reduction and refined estimation

As shown by the θ in Table 1, the model comprises 27 parameters in total, which are subjected to sampling input uncertainty using QMC and GSA. As mentioned above, eight parameters were initially found non-significant, the subjective input uncertainties of which are θ^lb^ ≤ θ ≤ θ^ub^, as shown in Table 1. In addition, the subjective input uncertainties of the rest 19 parameters are defined by the preliminary estimates θˆ and at the same time the values of *σ*^ex^ and *σ*^bg^ are restricted to be non-negative.

**Fig. 5:**
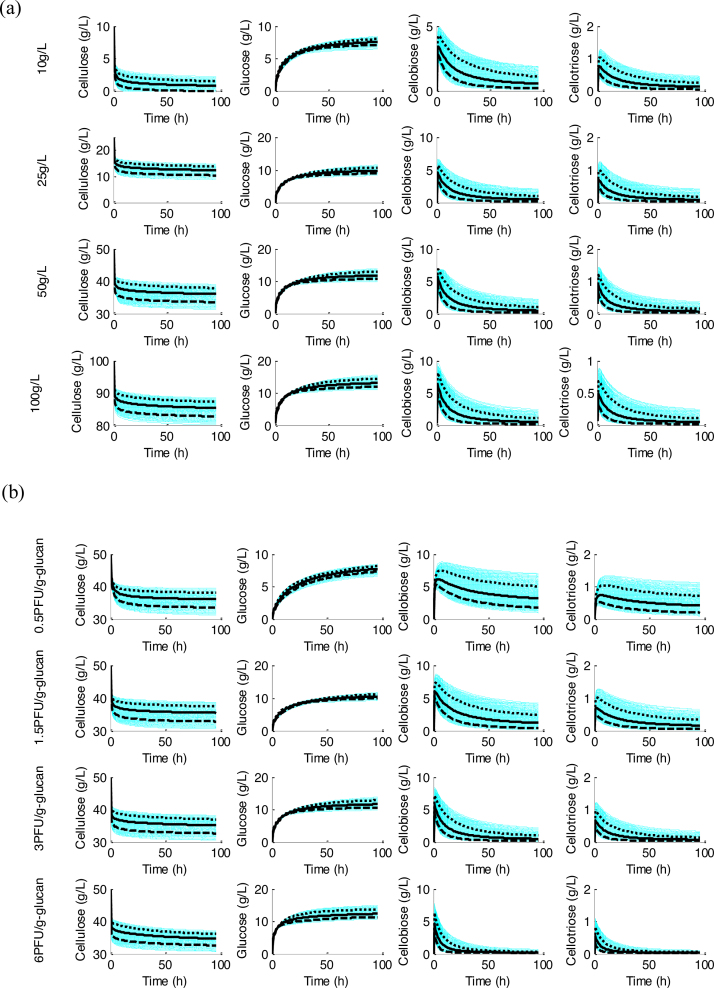
Representation of uncertainty of the model predications for cellulose, glucose, cellobiose, and cellotriose over hydrolysis with all the parameters (dim θ = 27). Quasi Monte Carle simulations (1000 samples, white blue lines), mean (→), and 10th () and 90th () percentile of the predictions. Typical cases: (a), starting non-crystalline cellulose concentrations of 10, 25, 50, and 100 g/L, and 6 FPU/g-glucan Spezyme^CP^ cellulases loading in all processes; (b), Spezyme^CP^ cellulases loadings of 0.5, 1.5, 3, and 6 FPU/g-glucan, and 50 g/L starting non-crystalline cellulose concentration in all processes.

At first, the QMC simulations obtained by simulating 1000 samples using the model result in 1000 time-series of dynamic profiles in Fig. 5 for a group of typical cases, i.e., starting cellulose concentrations of 10, 25, 50, 100 g/L and initial Spezyme^CP^ cellulases loadings of 0.5, 1.5, 3, 6 PFU/g-glucan. The model prediction uncertainty is represented using mean, 10th, and 90th percentile of the distribution of each model output at each time instant in Fig. 5. The prediction uncertainty of model outputs is illustrated by the spread of the prediction distribution, and the following conclusions can be obtained: (1) the aforementioned biphasic phenomena inevitably occurs with the hydrolysis processes in batch mode; (2) hydrolysis performance (i.e., the percent conversion of cellulose to soluble sugars) does not increase linearly but rather asymptotically with the increase of initial cellulases loadings; (3) interestingly, it is found that the major accumulation product is glucose in the all cases while the 90th percentile of cellobiose and cellotriose concentrations peaks around the end of the first (fast) hydrolysis phase (<10 g/L) and monotonically decreases thereafter.

**Fig. 6:**
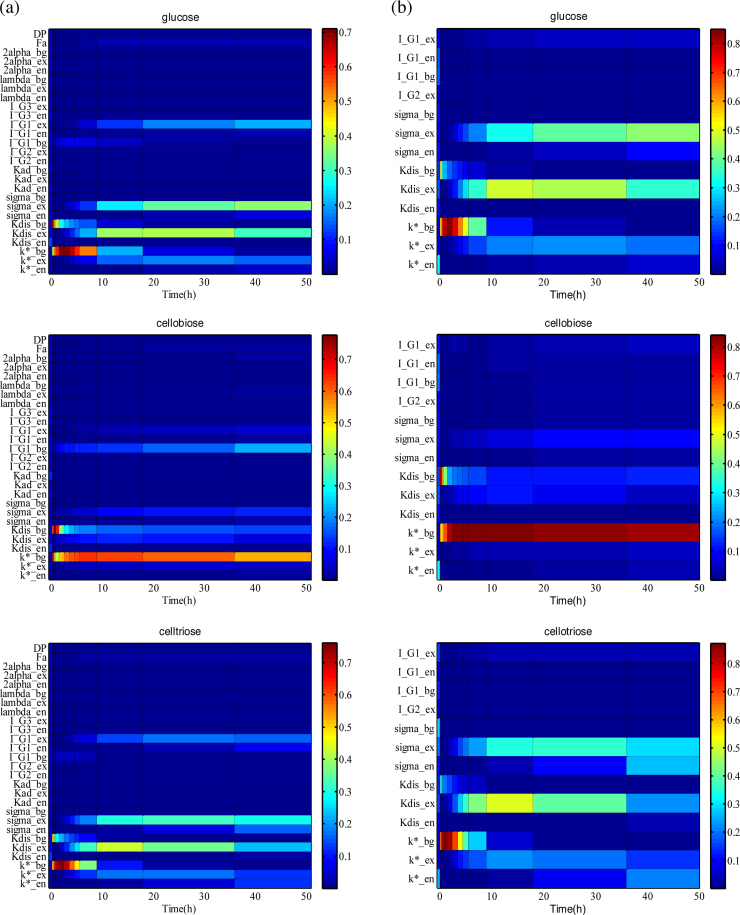
Sensitivities of model outputs (namely glucose, cellobiose, cellotriose from top to bottom) to 27 parameters (the column on the left hand, i.e., (a)) and 13 critical parameters (the column on the right hand, i.e., (b)) during the course of an example hydrolysis process (corresponding to Fig. 3(a)). Color axis scaling is given in every subplot. Only the values of total sensitivity indices ${S_{\theta_{i}}}^{tot}$ are shown.

Meanwhile, to understand what underlies this prediction uncertainty and what parameters are most critical to the model prediction, GSA is performed. The GSA results (Fig. 6(a)) illustrate that model outputs are insensitive to *F_a_*, *DP*, *λ*^*E*^ and 2*α*^*E*^over time in the given but reasonable spaces as noted in Table 1, based on a quantitative criterion of max($S_{\theta_{i}}^{\text{tot}}$) < 0.05 in this work. This means they can be fixed at the pre-selected values. In addition, it is found that other non-significant parameters are *K_ad_*^E^, *I_G2_*^en^, *I_G3_*^en^, and *I_G3_*^ex^. This can be explained as follows. Firstly, only low cellulases loadings (≤750 mg/L), i.e.,$\sum_{E=en,ex,bg}\left(E^{E}{}_{s,t}\text{+}E^{E}{}_{l,t}\right)$, were implemented experimentally and usually $E^{E}{}_{s,t}>3E^{E}{}_{l,t}$ (bound enzymes >75% w/w observed by [62], [63], [72], [78]). As a result, according to the corresponding parameter values in Table 1, it can be predicted that $\frac{1}{{K_{ad}}^{E}E^{E}{}_{l,t}}>3\times \frac{2\alpha^{E}}{M^{E}}$ under usual operating conditions. Consequently, Eq. (4) is transformed into $E^{E}{}_{s,t}≅\sigma^{E}{K_{ad}}^{E}E^{E}{}_{l,t}\sum_{i=4}^{N}\left[(i-1)G_{i}\right]$, i.e., only the products of $\sigma^{E}{K_{ad}}^{E}$ tend to be structurally identifiable under usual experimental conditions. Therefore, *K_ad_*^E^ can be set leaving just *σ*^E^ to be estimated. Secondly, thanks to the format of Michaelis–Menten kinetics and the fact that the preliminary estimates of *I*_G2_^en^, *I*_G3_^*en*^, and *I*_G3_^*ex*^ (shown by Table 1) are all much bigger than the corresponding product concentrations (the above-mentioned <10 g/L obtained by the QMC simulations), these three parameters can be practically eliminated from the model. As a result, a subset of 13 crucial parameters is identified.

The refined estimates for these parameters are summarized in Table 2 with the corresponding 95% confidence intervals. The corresponding correlation coefficients are shown in Fig. 4(b). It is found that the absolute values of all off-diagonal elements of correlation matrix are less than 0.6. From the ratios of $S_{\theta_{i}}$to ${S_{\theta_{i}}}^{\text{tot}}$(i.e.,$R_{\theta_{i}}$ in Eq. (41)) shown by Fig. S2, we can also conclude that the 13 parameters are less correlated unlike the very high interactions between some of the 27 parameters shown by Fig. S1. Additionally, as shown in Fig. 6 and Fig. S2, model predictions are generally more sensitive in the first 10 h of hydrolysis (i.e., the first phase), which means that more frequent sampling in this phase can yield more informative data for parameter estimation.

**Fig. 7:**
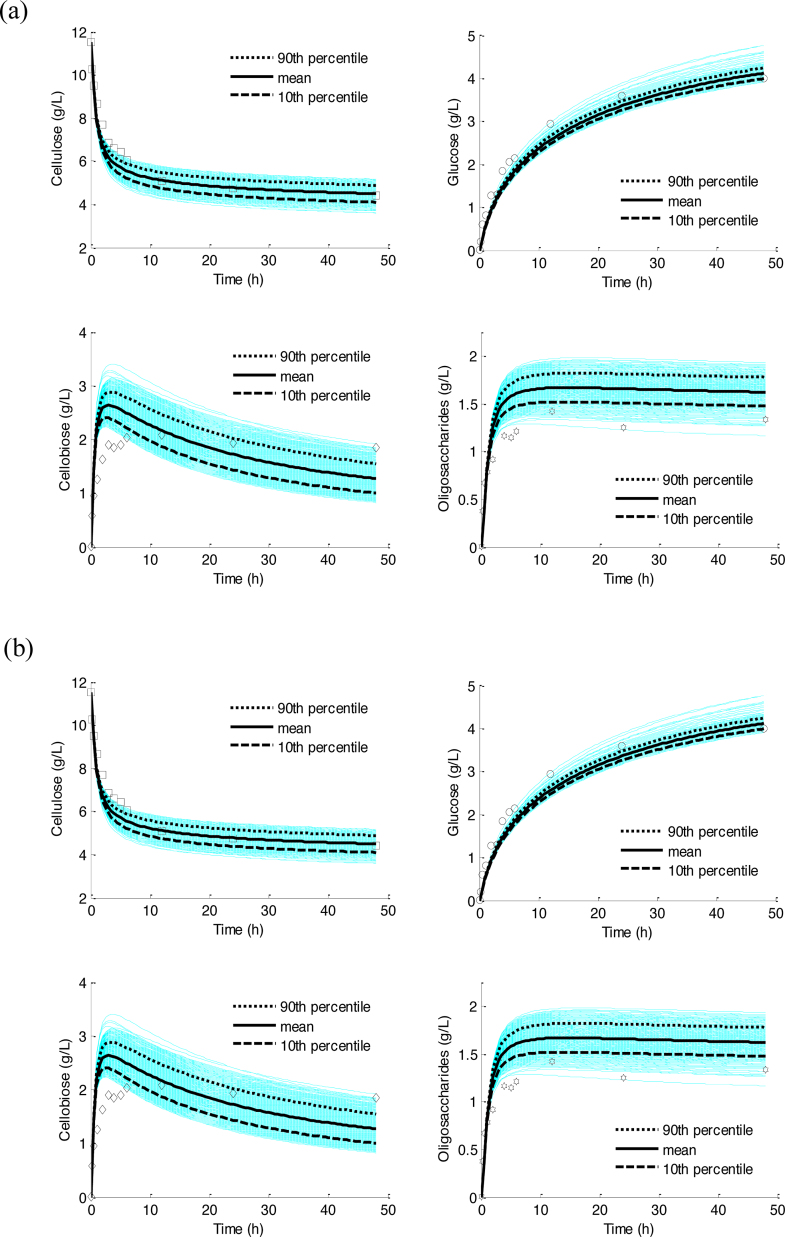
Representation of uncertainty of model predictions for cellulose, glucose, cellobiose, and cellotriose over hydrolysis with the reduced set of parameters (dim θ = 13). Quasi Monte Carle simulations (1000 samples, white blue lines), mean (→), and 10th () and 90th () percentile of the predictions. Two hydrolysis processes: (a), Spezyme^CP^ cellulases loading = 1 FPU/g-glucan; (b), Spezyme^CP^ cellulases loading = 3 FPU/g-glucan.

No significant difference is observed between the simulations with the reduced model of 13 crucial parameters (using the means) and with 19 parameters (using the means) (results not shown). By comparison, the J(θ) values change from 52.3 to 46.1 for the overall model and refined model, respectively. In addition, the uncertainty of model outputs is investigated by QMC simulations with the estimates of 13 parameters (Fig. 7 and Figure S3). Compared to Fig. 5, a smaller distribution spread of the outputs is observed in Fig. S4. Overall, as shown by the validation results (Fig. 6 and Fig. S3), the estimates are refined and the identifiability of the refined model is improved.

### 4.3 Model predictability and model-based optimisation

According to the results of the prediction uncertainty analysis, the model can be reduced to 13 crucial parameters with the other parameters eliminated or fixed at the values from literature under usual operating conditions. Note that the model should be carefully extrapolated to hydrolysis processes with high loadings of enzymes (>15 mg/g-glucan or >750 mg/L). Fortunately, high loadings are usually not used in industry applications due to high cost.

Besides direct validation with the data of Peri et al. [6], another set of data for phosphoric acid swollen cellulose (PASC, which was regarded as amorphous cellulose) hydrolysis was used for cross validation to check the model's predictive ability [44], [79]. As shown in Table 3, the model is indeed capable of predicting the experimental measurements with sufficient accuracy. This further suggests that the model can be successfully adapted to new processes.

**Fig. 8:**
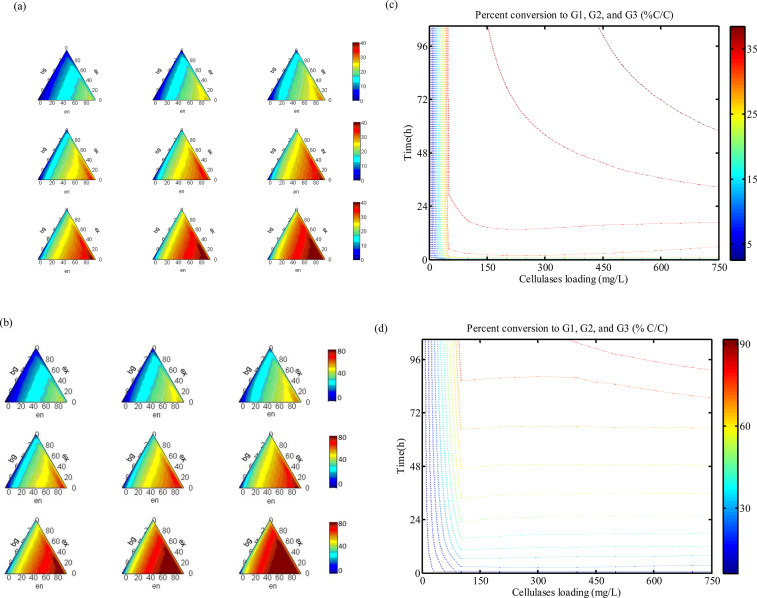
(a) and (b): Ternary plots of conversion rate $Y_{\mathrm{Eth/}G_{\mathrm{DP}}}$ (% C/C) as a function of varying total cellulase cocktail loadings and time during only hydrolysis (subplot (a)) and SSF (subplot (b)) of non-crystalline cellulose (both processes are of batch mode). In each subplot, from left to right, hydrolysis time of 24 h, 48 h, and 96 h; from top to bottom, total enzyme loadings of 2.5, 7.5, and 15 mg/g-glucan. (c) and (d): Contour plots of against time and cellulases loading at an optimum of en:ex:bg = 70:25:5(w/w/w) during only hydrolysis (subplot (c)) and SSF (subplot (d)) of non-crystalline cellulose (both processes are of batch mode). In all processes, the starting cellulose concentration is 50 g/L. The reaction temperature is 50 °C or 60 °C in only hydrolysis or in SSF, respectively. The values of k*^E^ are predicted by Arrhenius/van't Hoff equation ${k^{\text{E}}}_{\left(60{}^{\circ}C\right)}={k^{\text{E}}}_{\left(50{}^{\circ}C\right)}\text{exp}\left[-\frac{E_{\text{a}}}{R}\left(\frac{1}{60+273.15}-\frac{1}{50+273.15}\right)\right]$, where *E*_a_ is 47.6 kJ/mol [12] and ideal gas constant *R* is 8.314 J/K/mol.

This detailed mechanistic model provides a tool for optimising the cellulose hydrolysis process. An obvious target is to determine the optimal composition of cellulases cocktail including the three core constituents/enzymes. The optimised cocktail can, in theory, reduce the loading of enzymes without sacrificing the hydrolysis yield or increase the sugar yield in shorter incubation time. The optimisation procedure is as follows. Firstly, *in silico* simulations are performed under 1000 different make-ups of the three enzymes. Percent conversion $Y_{G_{123}/G_{N}}$ is calculated with respect to different compositions. Then, a ternary plot of the results clearly illustrates what compositions are optimal with respect to conversion to soluble sugars. Here, the “compositions” correspond to the samples that are taken for simulations. The optimisation is carried out for batch processes of both enzymatic hydrolysis and SSF. Initial cellulose concentrations are set to 50 g/L for both processes. As shown by subplots (a) and (b) of Fig. 8, it can be concluded that the optimal cocktail with the highest synergism should contain 60-85% endoglucanases, 10–30% exoglucanases, and 3–5% beta-glucosidase (w/w/w) for both processes. By comparison, the cocktail was experimentally optimized by Andersen et al. for the hydrolysis of PASC in batch mode [44], [79]. The optimal cocktail was found to be en:ex:bg = (50–100):(0–40):(10–40) (w/w/w). Gao et al. also experimentally optimized six core enzymes for saccharification of ammonia fiber expansion (AFEX) pretreated corn stover [80]. The optimal composition was found to be endo: CBH I: CBH II: beta-glucosidase: endo-xylanase: beta-xylosidase = 31.0:28.4:18.0:4.7:14.1:3.8 (w/w/w/w/w/w).

A reasonable enzyme loading would lie at a value between 2.5 and 7.5 mg/g-glucan as an activity-cost trade off (one could determine optimal enzymes loading by taking into consideration enzymes cost and increase in rate of hydrolysis). From the simulation results, in batch enzymatic hydrolysis, only around 40% of 50 g/L cellulose can be converted into soluble sugars by the optimal cellulases cocktail with 7.5 mg/g-glucan loading in 96 h. By comparison, about 90% of the cellulose is hydrolysed with the same cellulases cocktail in 96 h thanks to reduced inhibitions of products, which are simultaneously fermented into ethanol. According to subplots (c) and (d) of Fig. 8 that $Y_{G_{123}/G_{N}}$values are around 40% C/C and around 90% C/C after 96 h, respectively, using 375 mg/L (i.e., 7.5 mg/g-glucan) optimised cellulases cocktail.

As mentioned in the introduction, experimental validation using empirical models and pure mechanistic analysis of cellulose enzymatic hydrolysis (with high complexity, such as heterogeneous reaction, random chain and chain-end processive scission by different enzymes, and depolymerisation of polymer mixture) has typically been performed separately [12], [13], [14], [15], [16], [17], [18], [19], [20], [21], [22], [23], [24]. This work is an early stage effort to bridge the gap between detailed mechanistic modeling and experimental parametric studies. In the future, more process-level factors, such as diverse substrate physical properties as a result of various pretreatment methods, can be included into the model to make it more applicable to real processes. Following the systematic framework of parameter estimation and identifiability analysis presented herein (Fig. 2), model parameters can be identified and refined with experimental data available and/or using optimal experimental design. We aim to employ the developed model to guide the improvement of the hydrolysis and SSF processes in terms of operating conditions, initial cellulose concentrations, feeds of enzymes and cellulose, and separation of soluble sugars or ethanol. Additionally, the model can direct future rational approaches to the development of multi-gene expression systems to directly produce optimised enzyme mixtures of exoglucanase, endoglucanase, and beta-glucosidase [81], especially in the context of consolidated bioprocessing [82], [83], [84], [85].

## 5 Conclusions

In this work we developed a detailed mechanistic model of depolymerisation (chain-end scission) of amorphous cellulose by three core cellulolytic enzymes in a heterogeneous catalytic system. Separate steps for enzyme adsorption, formation of a catalytically active complex with cellulosic chains/ends, and enzyme desorption have been included. Through parametric analysis and experimental validation, we show that the resulting model with the reduced parameter set (13 out of an initial set of 27) is capable of predicting the evolution of distribution of insoluble cellulose chain lengths (from four to DP) as well as the production of soluble sugars during synergistic enzymatic hydrolysis. Finally, model-based optimisation for hydrolysis and SSF conclude that the optimal composition of cellulases cocktail is approximately en:ex:bg ≅ 60–85:10–30:3–5(w/w/w) both for enzymatic hydrolysis and for more advantageous SSF process.

## Figures and Tables

**Table 1:** Preliminary estimates of parameter values (dimθ = 19) with 95% confidence intervals at 50 °C.

θ	θ_refer_	θ^lb^	θ^ub^	θ_0_	$\hat{\theta }$	References
k*^en^	4.0	1.0	7.0	4.00	2.32 ± 0.16	[24], [75]
k*^ex^	0.80 ∼ 1.60	0.4	3.2	1.8	1.94 ± 0.23	[24], [75]
k*^bg^	0.193 ∼ 115	0.15	120	60.1	17.6 ± 1.20	[11], [73]
*k_dis_^en^*	0.333	0.1	2	1.05	1.22 ± 0.05	[24]
*k_dis_^ex^*	0.25	0.1	2	1.05	0.41 ± 0.03	[11], [24]
*k_dis_^bg^*	0.057 ∼ 4.81	0.03	10	5.02	8.46 ± 1.02	[71], [73], [74]
*σ^en^*	0.58 ∼ 1.9	0.2	10	5. 10	6.18 ± 2.51	[45]
*σ^ex^*	0.16 ∼ 0.75	0.05	10	5.03	5.81 ± 7.25	[45]
*σ^bg^*	0.269	0.1	5	2.55	0.47 ± 1.26	[61]
*k_ad_^en^*	15 ∼ 280	10	350	180	62.5 ± 3.2	[45], [61], [62], [72]
*k_ad_^ex^*	15 ∼ 280	10	350	180	44.2 ± 0.9	[45], [61], [62], [72]
*k_ad_^bg^*	15 ∼ 280	10	350	180	15.4 ± 2.7	[45], [61], [62], [72]
*I*_*G*2_*^en^*	0.015, 5.20	0.01	50	25	51.4 ± 6.48	[5], [6]
*I*_*G*2_*^ex^*	132, 5.20	0.5	150	75.3	2.15 ± 0.58	[5], [6]
*I*_*G*1_*^bg^*	0.07 ∼ 3.9	0.04	6	3	0.12 ± 0.09	[5], [6], [73], [74]
*I*_*G*1_*^en^*	0.1, 0.08	0.05	3	1.5	0.47 ± 0.21	[5], [6]
*I*_*G*1_*^ex^*	0.04, 0.08	0.02	3	1.5	0.82 ± 0.08	[5], [6]
*I*_*G*3_*^en^*	8.69	0.5	500	250	182 ± 32	[6]
*I*_G3_*^ex^*	8.69	0.5	500	250	295 ± 41	[6]
*F_a_*	0.12	0.09	0.16	0.12	Fixed at 0.12	[24]
*DP*	152	100	200	152	Fixed at 152	[24]
*λ^en^*,*λ^ex^*,*λ^bg^*	0.0122	0.006	0.0244	0.0122	Fixed at 0.0122	[37]
2*α^en^*/M*^en^*,						
2*α^ex^*/M*^ex^*,	1	0.5	1.5	1	Fixed at 1	[69], [70]
2*α^bg^*/M*^bg^*						

*Note*: some parameters were fixed at (1) *λ^en^* =* λ^ex^* = *λ^bg^* = 0.0122 h^−1^; (2) DP = 152, *F_a_* = 0.12; (3) 2*α^en^* = 43, 2*α^ex^* = 65, 2*α^bg^* = 110. Refer to Nomenclature for unit.

**Table 2:** Refined estimates of the parameter values for the reduced model (dimθ = 13) with 95% confidence intervals at 50 °C.

θ	θ_lb_	θ_ub_	θ_0_	$\hat{\theta }$	Unit
k^*en^	1.0	7.0	4.0	2.12 ± 0.04	10^3^ mmol/(g enzyme × h)
k^*ex^	0.4.0	3.2	1.8	1.52 ± 0.03	10^3^ mmol/(g enzyme × h)
k^*bg^	0.15	120	60.1	14.8 ± 0.5	10^3^ mmol/(g enzyme × h)
*K_dis_^en^*	0.10	2.0	1.05	1.28 ± 0.04	mmol β-glucosidic bonds/L
*K_dis_^ex^*	0.1	5.0	1.05	0.17 ± 0.02	mmol β-glucosidic bonds/L
*K_dis_^bg^*	0.03	10	5.02	6.58 ± 0.43	mmol β-glucosidic bonds/L
*σ*^*en*^	0.20	10	5.10	5.24 ± 0.68	10^−3^ mmol sites/mmolβ-glucosidic bonds
*σ*^*ex*^	0.05	10	5.03	6.25 ± 0.72	10^-3^ mmol sites/mmolβ-glucosidic bonds
*σ*^*bg*^	0.10	5.0	2.55	0.32 ± 0.21	10^-3^ mmol sites/mmolβ-glucosidic bonds
*I*_*G2*_^*ex*^	0.50	150	75.3	0.95 ± 0.24	g/L
*I*_*G1*_^*bg*^	0.04	6	3	0.11 ± 0.07	g/L
*I*_*G1*_^*en*^	0.05	3	1.5	0.78 ± 0.23	g/L
*I*_*G1*_^*ex*^	0.02	3	1.5	0.61 ± 0.14	g/L

*Note*: the pre-set values for the rest insensitive parameters are (1) *λ^en^* = *λ^ex^* =* λ^bg^* = 0.0122 h^−1^; (2) *DP* = 152, *F_a_* = 0.12; (3) 2*α^en^* = 43, 2*α^ex^* = 65, 2*α^bg^* = 110; (4) K_ad_^en^ = 50 L/mmol sites, K_ad_^ex^ = 450 L/mmol sites, K_ad_^bg^ = 15 L/mmol sites.

**Table 3:** Comparison between predictions and experimental measurements (shown in brackets and in red) of enzymatic hydrolysis products during degradation of PASC. (For interpretation of the references to colour in this Table 3, the reader is referred to the web version of this article.)

*Note*: n.d., not detected; d.l.a., detected in low amount; Comb., experimental combination number. (Experimental data from Andersen et al. [44], [79]).
