# Supplementary material for: Modelling of amorphous cellulose depolymerisation by cellulases, parametric studies and optimisation
Source: Biochem Eng J. 2016 Jan 15;105(Pt B):455–72. doi: 10.1016/j.bej.2015.10.017 (PMC4705870; doi:10.1016/j.bej.2015.10.017)
Supplement: Supplementary file 1 [file mmc1.docx]

(a) Sensitivities of glucose prediction with respect to parameters over time

$$S_{\theta_{i}}$$

$$S_{\theta_{i}}^{tot}$$

$$R_{\theta_{i}}$$

(b) Sensitivities of cellobiose prediction with respect to parameters over time

$$S_{\theta_{i}}$$

$$S_{\theta_{i}}^{tot}$$

$$R_{\theta_{i}}$$

(c) Sensitivities of cellotriose prediction with respect to parameters over time

$$S_{\theta_{i}}$$

$$S_{\theta_{i}}^{tot}$$

$$R_{\theta_{i}}$$

**Figure S1**. Sensitivities of model outputs (glucose (a) , cellobiose (b) , cellotriose (c) from top to bottom) to 27 parameters during the course of one hydrolysis process (as an example), which are $S_{\theta_{i}}$ $S_{\theta_{i}}^{tot}$and$R_{\theta_{i}}$ in sequence for each output.(a) Sensitivities of glucose prediction with respect to parameters over time

$$S_{\theta_{i}}$$

$$S_{\theta_{i}}^{tot}$$

$$R_{\theta_{i}}$$

(b) Sensitivities of cellobiose prediction with respect to parameters over time

$$S_{\theta_{i}}$$

$$S_{\theta_{i}}^{tot}$$

$$R_{\theta_{i}}$$

(c) Sensitivities of cellotriose prediction with respect to parameters over time

$$S_{\theta_{i}}$$

$$S_{\theta_{i}}^{tot}$$

$$R_{\theta_{i}}$$

**Figure S2**. Sensitivities of model outputs (glucose (a) , cellobiose (b) , cellotriose (c) from top to bottom) to 13 crucial parameters during the course of one hydrolysis process (as an example), which are $S_{\theta_{i}}$ $S_{\theta_{i}}^{tot}$and$R_{\theta_{i}}$ in sequence for each output.(a)

(b)

(c)

(d)

(e)

(f)

(g)

(h)

**Figure S3.** Representation of uncertainty of the model predications for cellulose, glucose, cellobiose, and cellotriose over hydrolysis with the reduced set of parameters (dim$\theta$=13). Quasi Monte Carle simulations (1000 samples, white blue lines), mean ( ), and 10^th^ ( ) and 90^th^ ( ) percentile of the predictions.

Cellulases loadings: 1 FPU/g-glucan in processes of a, b, c and d; 3 FPU/g-glucan in processes of e, f, g and h. Starting substrates: only noncrystalline cellulose in (a) and (e); noncrystalline cellulose with 5% (w/w) cello-oligosaccharides in (b) and (f); noncrystalline cellulose with 5% (w/w) glucose in (c) and (g); noncrystalline cellulose with 5% (w/w) cellobiose in (d) and (h).

(Data source: [6] Peri et al., Biotechnol. Prog. 23(3) (2007) 626-637.)

(a)

(b)

(b)

**Figure S4..** Representation of uncertainty of the model predications for cellulose, glucose, cellobiose, and cellotriose over hydrolysis with the reduced set of parameters (dim$\theta$=13). Quasi Monte Carle simulations (1000 samples, white blue lines), mean ( ), and 10^th^ ( ) and 90^th^ ( ) percentile of the predictions.

Typical cases: (a), starting non-crystalline cellulose concentrations of 10, 25, 50, and 100 g/L, and 6 FPU/g-glucan Spezyme^CP^ cellulases loading in all processes; (b), Spezyme^CP^ cellulases loadings of 0.5, 1.5, 3, and 6 FPU/g-glucan, and 50g/L starting non-crystalline cellulose concentration in all processes.
